# Reduced expression of gangliosides with GM2-determinant in cervical carcinoma-derived cells after subcutaneous transplantation into nude mice

**DOI:** 10.1007/s13577-023-00864-z

**Published:** 2023-03-17

**Authors:** Kyoko Tanaka, Isao Murakami, Mikio Mikami, Daisuke Aoki, Masao Iwamori

**Affiliations:** 1 Department of Obstetrics and Gynecology, Ohashi Hospital, Toho University, 2-22-36 Ohashi, Meguro-ku, Tokyo, 153-8515 Japan; 2 Department of Obstetrics and Gynecology, School of Medicine, Tokai University, 143 Shimokasuya, Isehara, Kanagawa 259-1193 Japan; 3 Department of Obstetrics and Gynecology, School of Medicine, Keio University, 35 Shinanomachi, Shinjuku-ku, Tokyo, 160-8582 Japan; 4 Animal and Human Model Project for Healthcare and Drug Development (Nomura Project), National Institutes of Biomedical Innovation, Health and Nutrition (NIBIOHN), 7-6-8 Asagi-Saito, Ibaraki, Osaka 567-0085 Japan

**Keywords:** Cervical carcinoma, Gangliosides, Subcutaneous transplantation, TLC-immunostaining

## Abstract

Human cervical carcinoma-derived cell lines have been frequently found to contain gangliosides with GM2-determinant, i.e., GM2, GalNAc-GM1b and GalNAc-GD1a, but GM2 was only detected in 5 of 15 tissues, and GalNAc-GM1b and GalNAc-GD1a were not found in any tissues from patients with several histological types of cervical carcinomas. To further characterize the ganglioside expression in cervical carcinomas, cells were grown by subcutaneous transplantation into nude mice, and gangliosides were quantitated by TLC-immunostaining with the anti-GM2 (YHD-06) antibody and a newly developed anti-GM3 (5H6) antibody, which reacts with GM3 and GM1b, but not with GD1a. Gangliosides with GM2-determinant in cells disappeared in transplanted cells, and the amount of GM3, a precursor for GM2, in transplanted cells was greater than in cultured cells. Also, transplanted cells containing GalNAc-GM1b newly expressed GM1b, suggesting that the activity of GalNAc transferase for synthesis of GalNAc-GM1b is retarded on subcutaneous transplantation. The ganglioside composition, with GM3 as the major one, in the transplanted cells was similar to that in cervical carcinoma tissues, and thus, the expression of gangliosides with GM2-determinant seemed to be accelerated under cell-cultivation conditions.

## Introduction

Glycosphingolipids are amphipathic molecules consisting of hydrophobic ceramide and hydrophilic carbohydrate moieties, and are ubiquitously distributed in tissues and cells. Due to aberrant expression of glycogenes in association with cellular transformation, their carbohydrate structures are frequently altered and are related to tumorigenicity, invasiveness, metastatic potential, drug resistance and immune evasion of cancer cells [1]. Although the expression of glycosyltransferase genes, among glycogenes, is particularly the significant for cancer-related carbohydrate antigens, their activities are regulated by ionic strength, pH, temperature, and the supplies of sugar nucleotides and precursor glycolipids in the multitransferase cascade for the synthesis of glycosphingolipids [2]. We previously reported that human uterine cervical carcinoma-derived cell lines contained unique gangliosides with GM2-determinant, e.g., GM2, GalNAc-GM1b and GalNAc-GD1a, in significantly high concentrations, amounting to 15.5–57.5% of the total gangliosides, which were not observed in ovarian and endometrial carcinoma-derived cell lines [3]. GM2 and GalNAc-GD1a, and GalNAc-GM1b belong to the ganglio-series a- and asialo-pathways, respectively, and their expression was not correlated with cell lines derived from different histological types of cervical carcinoma. In addition, GM2 was expressed in only 5 of 15 cervical carcinoma tissues and its concentration was less than 6% of the total gangliosides [3]. To further characterize glycolipid expression in cervical carcinomas, analysis was carried out using tissues obtained by subcutaneous transplantation of cells into nude mice.

## Materials and methods

### TLC-immunostaining of glycolipids

The glycolipids used in this experiment were purified from various sources in our laboratory according to the procedure described previously: GM3 from human erythrocytes, GM2, GalNAc-GM1b and GalNAc-GD1a from human brain from Tay–Sachs disease cases, GM1 and GD1a from bovine brain, and GM1b from rat ascite hepatoma cells [3]. Monoclonal antibodies to GM3 (5H6) and GM2 (YHD-06) were generated in our laboratory according to the conventional procedure. The antibodies and cholera toxin B-subunit (CTB) were utilized for quantitative determination of glycolipids by TLC-immunostaining [3].

### Human cervical carcinoma-derived cell lines

Squamous cell carcinoma (SCC)-derived SKG-IIIb, adenocarcinoma-derived HCA-1 and HeLa, and small cell carcinoma-derived HCSC-1 cells were purchased from RIKEN Bio Resource Center (Wako, Saitama, Japan), and the cells were injected subcutaneously into nude mice (KCN/SLC, Japan SLC, Hamamatsu, Shizuoka) and the resultant cell nodules were harvested 1–2 months after the injection. All animal experiments were performed in accordance with the guidelines for laboratory animal ethical procedures and were approved by the Animal Ethics Committee of Tokai University.

### Cervical carcinoma tissues

Human cervical carcinoma tissues were obtained from university hospitals, i.e., Tokai and Keio Universities. Written informed consent to use carcinoma tissues for this study was obtained from all subjects, and the experimental protocol was approved by the Ethics Committees of both hospitals. Histological classification was performed according to the criteria of the International Federation of Gynecology and Obstetrics [4].

## Results

### Monoclonal antibodies

**Fig. 1:**
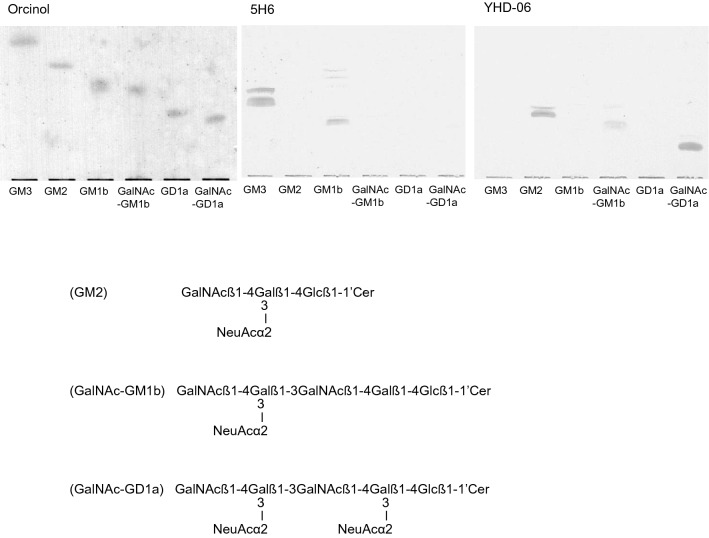
TLC and TLC-immunostaining of gangliosides. Gangliosides (0.5 μg) were developed on glass-coated TLC (orcinol) and plastic-coated TLC (5H6 and YHD-06) plates with chloroform/methanol/0.5% CaCl_2_ in water (55:45:10, by vol.), and the spots were visualized with orcinol-sulfuric acid reagent (orcinol), and anti-GM3 (5H6) and anti-GM2 (YHD-06) antibodies

As shown in Fig. 1, the monoclonal anti-GM2 (YHD-06) antibody reacted with GM2, GalNAc-GM1b and GalNAc-GD1a [3], whereas the anti-GM3 (5H6) one reacted not only with GM3, but also with GM1b, indicating that the NeuAcα2-3Gal-determinant is an epitope for the 5H6 antibody. Since GD1a did not react with the 5H6 antibody at all, the NeuAc-substituent at the second galactose residue of the gangliotetraose backbone was thought to interfere with the binding of the antibody with GD1a.

### Gangliosides in cultured and transplanted cervical carcinoma cells

**Fig. 2:**
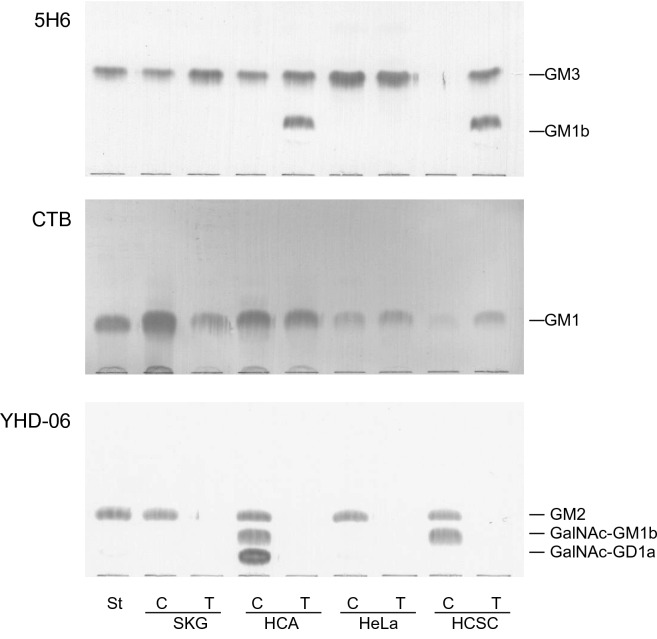
TLC-immunostaining with anti-GM3 (5H6) antibodies, CTB and anti-GM2 (YHD-06) antibodies of lipid extracts from cultured (C) and transplanted (T) cervical carcinoma-derived cells. Lipid extracts, corresponding to 0.1 mg for GM3 and GM2, and 0.01 mg for GM1, were developed with chloroform/methanol/0.5% CaCl_2_ in water (55:45:10, by vol.) and the spots were visualized with anti-GM3 antibodies (5H6), CTB (GM1), and anti-GM2 antibodies (YHD-06), respectively. St, standard glycolipids, 100 ng of GM3 and GM2, and 1 ng of GM1

As reported previously [3], all cells contained gangliosides with GM2-determinant at significantly high concentrations, e.g., GM2 alone in SKG-IIIb and HeLa cells, GM2 and GalNAc-GM1b in HCSC-cells, and GM2, GalNAc-GM1b and GalNAc-GD1a in HCA-1 cells. However, as shown in Fig. 2, gangliosides with GM2-determinant disappeared in the transplanted cells, although GM1, which was synthesized on transfer of terminal galactose to GM2, was detectable in the transplanted cells. It was noteworthy that GM1b was newly expressed in the transplanted HCA-1 and HCSC-1 cells, both of which contained GalNAc-GM1b, suggesting that the activity of GalNAc transferase for synthesis of GalNAc-GM1b is retarded in the transplanted cells. Similarly, although GM3 was not detected in the cultured HCSC-1 cells, it was present in the transplanted HCSC-1 cells, probably due to decreased synthesis of GM2, resulting in the accumulation of precursor GM3 in the transplanted HCSC-1 cells. The amounts of gangliosides in the cultured and transplanted cells are summarized in Table 1. The amounts of GalNAc-GM1b in the cultured HCA-1 and HCSC-1 cells were comparable to those of GM1b in the transplanted HCA-1 and HCSC-1 cells, respectively, indicating that the synthetic potential of GM1b is maintained in the transplanted cells. Whereas, the amounts of GM2 in the cultured cells were not clearly correlated with those of GM3 in the transplanted cells. On the other hand, expression of GM1 was maintained at relatively constant levels in the cultured and transplanted cells.

**Table 1:** Amounts of gangliosides in the cultured and the transplanted cervical carcinoma-derived cells (µg/mg dry weight)

	SCC-derived SKG-IIIb		Adeno-derived HCA-1		Adeno-derived HeLa		Small cell-derived HCSC-1	
	Culture	Trans	Culture	Trans	Culture	Trans	Culture	Trans
GM3	0.17 ± 0.07	0.48 ± 0.19	0.18 ± 0.02	0.40 ± 0.05	0.87 ± 0.11	0.62 ± 0.12	–	0.31 ± 0.08
GM2	0.13 ± 0.05	–	0.15 ± 0.02	–	0.12 ± 0.01	–	0.10 ± 0.01	–
GM1	0.22 ± 0.02	0.11 ± 0.01	0.14 ± 0.01	0.11 ± 0.01	0.04	0.05	0.01	0.05
GM1b	–	–	–	0.22 ± 0.02	–	–	–	0.25 ± 0.02
GalNAc-GM1b	–	–	0.18 ± 0.03	–	–	–	0.19 ± 0.01	–
GalNAc-GD1a	–	–	0.28 ± 0.06	–	–	–	–	–

*SCC* squamous cell carcinoma, *Adeno* adenocarcinoma, *Small cell* small cell carcinoma, *Culture* cultured cells, *Trans* transplanted cells

### Gangliosides in the tissues of patients with several types of cervical carcinoma

**Fig. 3:**
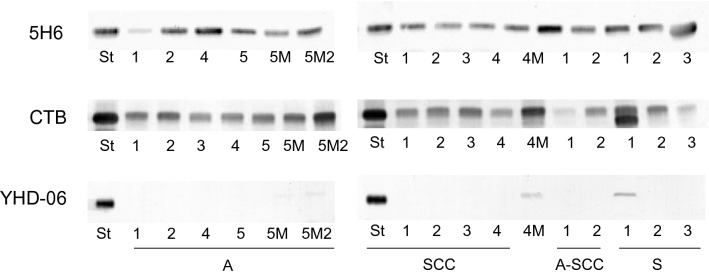
TLC-immunostaining with anti-GM3 (5H6) antibodies, CTB and anti-GM2 (YHD-06) antibodies of lipid extracts from the tissues of patients with several types of cervical carcinoma. A, adenocarcinoma; SCC, squamous cell carcinoma; A-SCC, adenosquamous carcinoma; S, small cell carcinoma; 1–5, patient numbers; 5M1 and 5M2, and 4 M, metastatic tissues in the lymph nodes of A-patient 5 and SCC-patient 4, respectively; St, standard glycolipids, 100 ng of GM3 and GM2, and 1 ng of GM1. The procedure was the same as described in the legend to Fig. 2

Ganglioside expression in the tissues of patients was determined by TLC-immunostaining with anti-GM3 antibodies (5H6), CTB and anti-GM2 antibodies (YHD-06), as shown in Fig. 3. Although GM3 and GM1 were ubiquitously expressed in all tissues examined, GM2 was present in only three out of 14 tissues in amounts of less than 0.02 μg/mg dry weight, e.g., SCC-patient 4 and the metastatic tissues, adenocarcinoma-patient 5 and metastatic tissues, and small cell carcinoma-patient 1. The major ganglioside, amounting to more than 84% of total gangliosides, was GM3, and GM1 was in amounts of 0.01–0.06 μg/mg dry weight, i.e., less than 12% of the total gangliosides. GM1b, GalNAc-GM1b and GalNAc-GD1a were undetectable, even on examination of the lipid extracts, corresponding to 1 mg dry tissue weight.

## Discussion

The ubiquitous expression of GM3 as the major ganglioside and of GM1 as a minor one in the tissues of patients with cervical carcinomas resembled that in the nodules formed on subcutaneous transplantation into nude mice. Accordingly, enhanced synthesis of gangliosides with GM2-determinant was thought to be an event occurring under cell-cultivated conditions for cervical carcinoma-derived cells, but not for ovarian and endometrial carcinoma-derived cells. As reported in our previous paper [3], gene expression of GalNAc-transferase (B4galnt 1) was closely associated with high expression of GM2, GalNAc-GM1b and GalNAc-GD1a in cultured cervical carcinoma-derived cells, but their syntheses seemed to be dependent on the supplies of precursor glycolipids and sugar nucleotides at the individual metabolic steps in the multitransferase cascade. In fact, when the ganglioside compositions of seven cervical carcinoma-derived cells examined were compared, although GM2 was found in all cells, GalNAc-GM1b and GalNAc-GD1a were detected in four and three out of seven cell lines, probably mainly due to supply of GM1b in the ganglio-series asialo-pathway, and of GD1a in the ganglio-series a-pathway, respectively [2]. Apparently, the basic metabolic pathways seemed to be affected by the circumstances surrounding cells in vitro and in vivo to give the characteristic glycolipid compositions in cultured and transplanted cells, and within the human body. The activities of papilloma viruses (PV)-16 and -18, which are responsible for carcinogenesis of cervical cells, were also reported to affect the lipid compositions including glycerophospholipids and glycosphingolipids on lipidomic analysis, the difference between PV-16 and PV-18 being favorable and poor prognoses, respectively [5]. Since enhanced synthesis of GM2 has been reported in fibroblast and neural cells after infection with SV-40 virus [6], the ubiquitous high expression of gangliosides with GM2-determinant in cervical carcinoma-derived cells was thought to be due to infection with PV [3]. Since there is a report that PV in cervical carcinoma patients disappeared in their cancerous tissues and sera, analyses of PV in transplanted tissues and human tissues is now in progress in our laboratory.
